# Cigarette Smoking Is Negatively Associated with the Prevalence of Type 2 Diabetes in Middle-Aged Men with Normal Weight but Positively Associated with Stroke in Men

**DOI:** 10.1155/2019/1853018

**Published:** 2019-09-11

**Authors:** Su Wang, Jie Chen, Yuzhong Wang, Yu Yang, Danyu Zhang, Chao Liu, Kun Wang

**Affiliations:** ^1^Department of Endocrinology, Affiliated Jiangning Hospital of Nanjing Medical University, Nanjing, China; ^2^Department of Endocrinology, Jiangsu Province Hospital on Integration of Chinese and Western Medicine, Nanjing University of Chinese Medicine, Nanjing, China; ^3^Department of Geriatrics, Affiliated Jiangning Hospital of Nanjing Medical University, Nanjing, China

## Abstract

**Background:**

The prevalence of diabetes and potentially related complications, including stroke, is rapidly increasing in China. The long-term effects of lifestyle may affect glucose metabolism in the general population. Although some studies have shown an association between smoking and the risk of type 2 diabetes mellitus (T2DM), the relationship remains unclear. Furthermore, the relationship between smoking and stroke in patients with T2DM has not been fully elucidated.

**Objective:**

We investigated the influence of cigarette smoking on T2DM and stroke in China. Detailed questionnaires about smoking status and anthropometric measurement were completed by participants, and oral glucose tolerance testing (OGTT), hemoglobin A1c (HbA1c), homeostasis model assessment of IR (HOMA-IR), and blood lipids were measured.

**Results:**

In total, 8196 adults aged 40 years or older were included. We found a reduced risk of impaired glucose regulation (IGR) and T2DM in male smokers with normal weight (body mass index (BMI) < 25 kg/m^2^ or waist circumference (WC) < 90 cm) compared with nonsmokers after adjusting for age, alcohol intake, physical activity, educational level, family history of diabetes, SBP, DBP, TG, TC, HDL-C, and LDL-C. However, no significant association was detected in male current smokers with overweight/obesity or female smokers. In addition, compared with nonsmokers without T2DM, current smokers with T2DM had a significantly higher risk of stroke (odds ratio: 2.64, 95% confidence interval: 1.25–5.57; *P* = 0.011) after adjusting for confounders.

**Conclusions:**

Smoking was negatively associated with T2DM in Chinese men of normal body weight, but no significant association was found for men with overweight/obesity or women. In addition, smoking was positively associated with nonfatal stroke, especially in patients with T2DM. Further prospective studies are needed to examine the association between smoking, diabetes, and stroke in different ethnic groups.

## 1. Introduction

Smoking is a leading risk factor for noncommunicable diseases (NCDs), such as cardiovascular disease [1, 2], cancer [3, 4], and chronic respiratory diseases [5]. Although the rate of smoking has fallen in many high-income countries in recent years, the number of smokers in China increased from 300 million to 315 million (around 28% of the adult population) between 2010 and 2015. More than one million people die in China every year due to tobacco use, and on this trajectory, the total number is expected to rise to three million annually by 2050 [6]. Diabetes represents an increasing public health burden in China, with the current prevalence estimated at 11.6% while the prevalence of prediabetes is approximately 50.1% [7]. Unfortunately, stroke also remains a major public threat, with recent national data showing that the age-standardized prevalence and mortality rates have reached 1114.8/100,000 people and 114.8/100,000 person-years [8]. Diabetes is a well-established risk factor for macrovascular clinical complications, including stroke [9]. A meta-analysis of 102 prospective studies showed that the adjusted hazard ratios (HRs) for diabetes were 2.27 (1.95–2.65) for ischemic stroke and 1.56 (1.19–2.05) for hemorrhagic stroke [10]. China is thus on the verge of being overwhelmed by the prevalence of diabetes and stroke, which are associated with a heavy socioeconomic burden [11, 12].

The relationship between smoking and type 2 diabetes mellitus (T2DM) is unclear. Although most epidemiological evidence has demonstrated a positive association between smoking and T2DM [13–15], several studies found no significant association or even a negative relationship between the two of them [16–19]. Considering the differences in races and significant heterogeneity across the studies performed to date, the application of these conclusions remains limited. Further high-quality studies are thus needed to explore the relationship between smoking and diabetes in specific populations. We therefore sought to investigate the effect of cigarette smoking on T2DM in a Chinese population and to evaluate smoking amount and adiposity as potential effect modifiers. Furthermore, the risk of stroke associated with smoking in patients with T2DM was examined.

## 2. Subjects and Methods

### 2.1. Study Population

The Risk Evaluation of Cancers in Chinese Diabetic Individuals: a community-based longitudinal (REACTION) study was a multicenter prospective observational study of adults aged 40 years or older. The primary purpose of the study was to assess the relationship between abnormal glucose metabolism and the risk of cancer in the Chinese population. Details of the study design have been published previously [20]. The data presented in the current analysis were based on a baseline survey of subsamples from Maigaoqiao community of Nanjing, a subcenter of Jiangsu Province, between June 1, 2011 and November 31, 2011. This study was approved by the Institutional Review Board of Shanghai Jiaotong University. Informed consent was obtained from all participants prior to participation.

### 2.2. Data Collection

All participants were interviewed by qualified health employees using face-to-face standardized questionnaires. Data collected included educational level, smoking status (including duration of smoking history or since quitting smoking), alcohol consumption, physical activity, and medical history (including current medications and diseases such as diabetes mellitus, hypertension, and stroke). A total of 8196 adults aged ≥40 years old were included in this analysis. Enrolled subjects who did not complete oral glucose tolerance testing (OGTT) or provided incomplete questionnaire information were excluded from the analysis. According to the theory of obesity paradox, participants with a body mass index (BMI) < 18.5 kg/m^2^ were excluded to eliminate the effect of morbidly lean patients on diabetes [21]. Individuals with type 1 diabetes, moderate to severe hepatic/renal dysfunction, hyperthyroidism, hypothyroidism, or cancer were excluded from the analysis, as were subjects who had ever quitted smoking but smoked again, or who quitted smoking repeatedly. Only cigarette smokers were included, and subjects who favored the use of hand-rolled cigarettes or hookah were excluded. Those who favored smoking both cigarettes and other kind of tobacco were also excluded, as were subjects who could not provide a detailed smoking status.

All included individuals were categorized into nonsmokers, exsmokers, and current smokers. Those who had never smoked or smoked less than 100 cigarettes in their lifetime were considered nonsmokers, those who had regularly smoked previously but had quit smoking >1 year prior to the interview were considered exsmokers, and those who currently smoked at least one cigarette per day or seven cigarettes per week on a regular basis, as well as those who had been quitting smoking <1 year prior to the interview were defined as current smokers. Exsmokers were asked how long ago they had stopped smoking. Current smokers were asked the age at which they started smoking, years of smoking, and number of cigarettes smoked per day. Smoking amount was defined according to the number of cigarettes per day (light: 1–19; heavy: ≥20) or pack-years (light: 1–19; heavy: ≥20). Pack-years were calculated as follows: packs (one pack = 20 cigarettes) smoked per day × years of smoking.

Educational level was classified as low (no education, primary education, or junior middle education), medium (high-school education), or high (college education). Regular leisure-time physical activity was defined as participation in moderate or vigorous activity for more than 30 minutes per day at least three days a week. The diagnosis of nonfatal stroke was based on subjects' self-reporting and previous medical history. All subjects who stated a history of stroke were required to state the hospital where they were diagnosed or provide evidence of disease diagnosis.

Anthropometric measurements including height, weight, waist circumference (WC), and hip circumference were performed using a standard protocol. BMI was calculated as weight divided by height squared. The waist to hip ratio (WHR) was calculated as the ratio of waist and hip circumference.

All participants underwent an OGTT and provided blood samples which were tested for fasting plasma glucose (FPG), and 2-hour postprandial plasma glucose (2hPG), fasting insulin (FINS), hemoglobin A1c (HbA1c), total cholesterol (TC), triglyceride (TG), high-density lipoprotein cholesterol (HDL-C), and low-density lipoprotein cholesterol (LDL-C) according to China National Laboratory Accreditation [20]. To estimate insulin sensitivity and beta cell function, the homeostasis model assessment (HOMA) was used: HOMA‐IR = FINS (mU/L) × FPG (mmol/L)/22.5, HOMA‐*β* = 20 × FINS (mU/L)/[FPG (mmol/L)–3.5].

### 2.3. Definition of T2DM

All participants were required to fast for at least 10 hours before undergoing a 75 g OGTT. Diagnosis of normal glucose tolerance (NGT), prediabetes, and T2DM was based on the 2017 T2DM diagnostic criteria of the Chinese Diabetes Society (CDS) [22]. NGT was defined as FPG < 6.1 mmol/L and 2hPG < 7.8 mmol/L, prediabetes was defined as impaired fasting glucose (IFG) (FPG ≥ 6.1 and <7.0 mmol/L) and/or impaired glucose tolerance (2hPG ≥ 7.8 and <11.1 mmol/L), and T2DM was defined as FPG ≥ 7.0 mmol/L, and/or 2hPG ≥ 11.1 mmol/L, and/or previous diagnosis of T2DM.

### 2.4. Statistical Methods

All statistical analyses were conducted using SPSS version 23.0 (SPSS Inc., Chicago, IL). We first conducted analysis of variance (ANOVA), the Kruskal-Wallis test (as appropriate), or the *χ*^2^ test to draw an overall *P* value of comparison of three groups and then do the appropriate post hoc pairwise comparisons. The *P* values were corrected with the Bonferroni correction by multiple comparison between each status of glucose metabolism. Multinomial multivariable logistic regression was performed to assess the association between smoking status and glycemic regulation status (prediabetes, T2DM) using the Enter method. Multivariable binary logistic regression was conducted to obtain the odds ratio (OR) with 95% CIs (confidence intervals) for the association of smoking exposure and T2DM with stroke. A two-tailed test was used, and *P* values of <0.05 were regarded as statistically significant.

## 3. Results

### 3.1. Characteristics of Study Participants

The clinical baseline characteristics of the total study population and the subgroups studied are summarized in Table 1. Compared with NGT subjects, age, BMI, WC, WHR, FPG, 2hPG, HbA1c, TG, TC, FINS, HOMA-IR, and SBP were significantly increased in the T2DM group, while HDL-C and HOMA-*β* were significantly decreased. Subjects with T2DM had lower frequencies of physical activity and family histories of diabetes compared with the NGT group. The proportion of current smokers was 18.9%, 15.8%, and 16.3% in the NGT, prediabetes, and T2DM groups, respectively. The proportion of patients with nonfatal stroke was significantly higher in the T2DM group than in the NGT group. A comparison of baseline data between men and women is shown in Supplementary [Supplementary-material supplementary-material-1].

### 3.2. Association between Smoking Status and IGR and T2DM

After adjustment for age, BMI, gender, educational level, physical activity, alcohol consumption, family history of diabetes, SBP, DBP, TG, TC, HDL-C, and LDL-C, current smokers were associated with a 23% (OR: 0.77, 95% CI: 0.63–0.93) and 20% (OR: 0.80, 95% CI: 0.66–0.98) decreased risk of prediabetes and T2DM compared with nonsmokers, respectively.

In addition, light smokers (1–19 cigarettes/day or 1–19 pack-years) exhibited a lower risk of having T2DM, while no significant association with risk of T2DM was found for heavy smokers and nonsmokers (Table 2). The odds of exhibiting IGR were lower among both light and heavy smokers compared with nonsmokers (Supplementary [Supplementary-material supplementary-material-1]).

### 3.3. Association between Cigarette Smoking and IGR/T2DM Stratified by BMI, WC, and WHR

In men, stratification by normal weight (BMI < 25 kg/m^2^, WC < 90 cm, and WHR < 1.0) showed that light and heavy smokers had a lower risk of prediabetes/T2DM than nonsmokers. Current smokers with overweight/obesity (BMI ≥ 25 kg/m^2^, WC ≥ 90 cm, and WHR ≥ 1.0) also had no significant association with IGR or T2DM (Table 3 and Supplementary [Supplementary-material supplementary-material-1]). Given the low smoking rate in women included in the study population, a stratified analysis according to smoking amount or BMI was not performed. With nonsmokers used as a reference, the corresponding OR (95% CI) for abnormal glucose metabolism (including prediabetes and T2DM) was 1.34 (0.80–2.24) in current smokers (*P* = 0.267) after adjustment for confounders (including age, gender, BMI, educational level, physical activity, alcohol consumption, family history of diabetes, SBP, DBP, TG, TC, HDL-C, and LDL-C).

### 3.4. Association between Cigarette Smoking and Obesity and HOMA-IR

**Figure 1:**
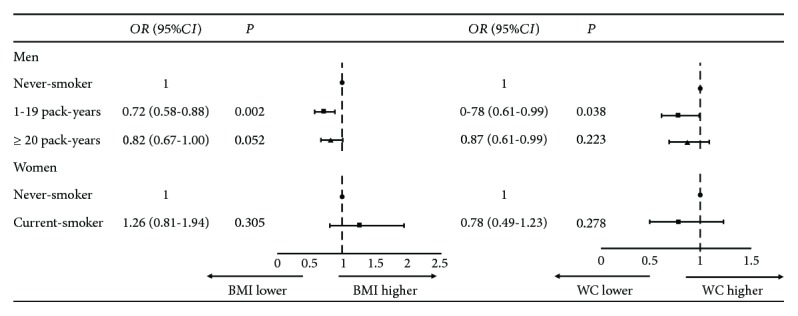
Association between smoking status and overweight/obesity (*N* = 7904). Adjusted for age, educational level, physical activity, and alcohol consumption status.

Overall adiposity was defined as BMI ≥ 25 kg/m^2^. Central adiposity was defined as WC ≥ 90 cm in men and ≥85 cm in women. In men, the risk of overweight/obesity (both BMI ≥ 25/kg^2^ and WC ≥ 90 cm) was significantly reduced in light smokers after adjustment for confounders. However, no significant association was found between smoking status and obesity in women (Figure 1).

**Figure 2:**
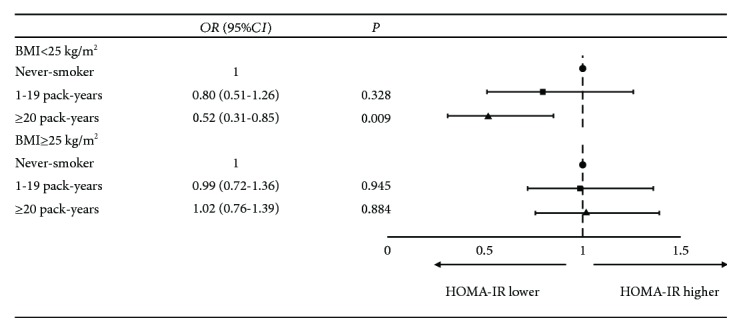
Association between smoking status and insulin resistance in men stratified by BMI (*n* = 2494). Adjusted for age, educational level, physical activity, and alcohol consumption status.

Individuals with HOMA-IR in the highest quartile were defined as insulin resistant. Using nonsmokers as the reference group, multivariable regression analysis in men showed that heavy smokers with BMI < 25 kg/m^2^ were less likely to have insulin resistance (Figure 2). In women, the corresponding adjusted OR (95% CI) for insulin resistance was 1.22 (0.74–2.02) in current smokers.

### 3.5. Interaction between Smoking and T2DM in relation to Stroke

The prevalence of self-report stroke was 2.3% in our study. No significant difference was observed in the prevalence of stroke between men and women (2.4% vs. 2.2%, *P* = 0.599). The association of stroke with an interaction term (between smoking status and T2DM) was significant (*P* = 0.023), after adjusting for confounders. Using nonsmokers without diabetes as a reference, current smokers with T2DM had a significantly higher risk of stroke (OR: 2.64, 95% CI: 1.25–5.57; *P* = 0.011) after adjusting for confounders (Table 4).

## 4. Discussion

Smoking is an established major risk factor for various chronic noncommunicable diseases [23–25], although evidence for a relationship between smoking and diabetes remains controversial [14, 16]. Although the majority of studies have demonstrated that smoking may increase the risk of diabetes [13–15], some evidence has pointed to a decreased risk [16, 19]. The China National Diabetes and Metabolic Disorders Study analyzed data from 16,286 men and found a negative association between smoking and newly diagnosed diabetes as identified by OGTT in males with a normal body weight (BMI 18.5–25 kg/m^2^) [16]. Data for over 12,000 American adults who participated in the National Health and Nutrition Examination Survey (NHANES) 2005–2014 showed that smokers were less likely to have T2DM than nonsmokers (OR: 0.41; 95% CI: 0.35–0.48) [19]. Similar results were reported in a prospective study of 3385 participants in Turkey during a mean 5.9-year follow-up, whereby individuals who smoked >10 cigarettes per day (mean BMI 26.4 kg/m^2^) had an adjusted relative risk (RR) (with adjustment for age, baseline family income bracket, and physical activity grade) for diabetes of 0.54 (95% CI: 0.35–0.83) as compared with nonsmokers (mean BMI 28.2 kg/m^2^) in all adults [18]. In a follow-up study over a mean period of 7.4 years, incident diabetes occurred in 69 men out of 16,829 apparently healthy men. After adjustment for age, alcohol consumption, exercise level, and education, light smoking (<20 cigarettes/d) reduced the risk of diabetes in lean men (BMI in the lowest quartiles, BMI < 21.3 kg/m^2^) [26].

Similar to the studies described above, our findings show that cigarette smoking was associated with decreased odds of T2DM in men with BMI < 25 kg/m^2^ compared with those who did not smoke. We also explored the relationship between smoking status and HOMA-IR and observed that an improvement of insulin resistance was only found in current smokers with BMI < 25 kg/m^2^. This association was not observed in male smokers with BMI ≥ 25 kg/m^2^, which supports data from the China National Diabetes and Metabolic Disorders Study in which no association was identified between smoking and newly diagnosed diabetes in males who were overweight/obese (BMI ≥ 25 kg/m^2^) [16]. These differences in results when classified by BMI/WC indicate that obesity category should be taken into consideration when evaluating the relationship between diabetes and smoking.

Given the different relationships identified between smoking and T2DM in different BMI/WC groups among men, we explored the relationship between smoking and obesity in the present. Logistic regression revealed significant negative associations between smoking and obesity in males, but no such association was found in women. Similarly, a previous study in Japan found that male current smokers had a lower OR for obesity compared with nonsmokers [27]. However, another study found a linear increase in the OR of abdominal obesity with increasing levels of both smoking status and amount in T2DM [28]. In addition, the relationship between smoking status and obesity risk has been found to differ by age. In that study, smoking was shown to increase obesity among young men, but to decrease obesity among middle-aged and older men [29]. To date, the relationship between smoking and obesity remains unclear, and further studies are clearly needed.

The inconsistent results between smoking and T2DM/obesity identified in our study were also found between men and women, as our data show that there was no evidence for an association between smoking and T2DM/obesity in women. The results of the Japanese study described above also showed that there were no differences in obesity between current smokers and nonsmokers [27]. The MONICA/KORA Augsburg Cohort Study in 5422 women in Germany also showed that there was no association between smoking and diabetes [30]. The inconsistency in results between men and women might be related to differences in smoking density and intensity. In China, specific sociocultural characteristics mean that the overall smoking rate among women is clearly lower than that among men [31]. In our study, only 1.6% of women were current smokers, significantly lower than the 48.8% of men. Furthermore, the mean pack-years of smoking in male current smokers was twice as high for men than for women (median: 20 vs. 10, *P* < 0.001).

Differences between our findings and those reported in several previous studies might in part be attributable to the following factors. First, the BMI cutoff points to define overweight and obesity were different in different studies. In addition, some studies used measures other than BMI to define obesity, including WC, WHR, visceral fat thickness, fat percentage, and fat mass. These differences in the method used to estimate obesity may have led to differences in the results. Second, the participants enrolled in the present study were all Chinese, so we used the CDS diabetes diagnostic criteria. The FPG levels for IFG were higher in the 2017 CDS diagnostic criteria compared with the diagnostic criteria of the American Diabetes Association used in other studies. Third, some other studies used only self-reported data, while our study combined medical histories and standard OGTT results to diagnose T2DM. Finally, the inclusion of different patient populations in the various studies might have contributed to population bias, thus affecting the study conclusions to some extent. Further studies of the relationship between smoking and obesity in patients with diabetes and the possible mechanism responsible for such a relationship are therefore required.

Diabetes is an established major risk factor for stroke [32]. Of note, although the risk of prediabetes/T2DM was lower in current smokers who were lean men, our findings also showed that smoking was an independent risk factor for stroke after adjustment for confounders. Our study also showed that current smokers with T2DM had a significantly higher risk for stroke compared with patients who only had T2DM or only smoked, after adjustment for confounders. Evidence for a beneficial effect on subsequent stroke attributable to a reduced risk of abnormal glucose metabolism was not identified in current smokers.

This study had several limitations. First, although we enrolled 5422 women, there were only 87 current smokers in this group. The limited data might have affected the reliability of our conclusions for women. Further studies with a focus on women are thus required. Second, 27.1% of T2DM patients had received antidiabetic drugs, which might have affected the accuracy of the measurement of insulin levels and further affect HOMA-IR. Third, the diagnostic criteria for stroke were assessed based on patient self-reported data and medical history (although they provided evidence of disease diagnosis), which might have resulted in potential bias in the prevalence of stroke. Fourth, all subjects were of Chinese ethnicity, meaning that the results of the study might not be applicable to other populations. Finally, the use of a cross-sectional design means that it is not possible to infer a causal relationship between smoking status and prediabetes/T2DM. Prospective studies with larger sample sizes in specific populations are thus needed to clarify the relationship between smoking behavior and diabetes, related complications, and total morality.

## 5. Conclusions

The results of this cross-sectional study indicate that smoking is negatively associated with IGR/T2DM in Chinese men with normal body weight, although no significant association was found for overweight/obese Chinese men or for women. In addition, smoking and T2DM had a combined positive correlation with stroke after adjusting for confounders (including BMI). Therefore, smoking cessation should be suggested to all current smokers, regardless of BMI.

## Figures and Tables

**Table 1:** Characteristics of the population according to the status of glucose metabolism (*N* = 8196).

Variables	NGT (A) *n* = 4399	Prediabetes (B) *n* = 1864	T2DM (C) *n* = 1933	*P* overall	*P*-B vs. A	*P*-C vs. A
Age (years)	55.91 ± 10.06	61.16 ± 9.97	63.01 ± 9.65	**<0.001**	**<0.001**	**<0.001**
Sex, male/female (*n*)	1396/3003	637/1227	741/1192	**<0.001**	0.177	**<0.001**
BMI (kg/m^2^)	24.45 ± 3.27	25.26 ± 3.50	25.27 ± 3.67	**<0.001**	**<0.001**	**<0.001**
WC (cm)	87.90 ± 9.09	90.16 ± 9.12	90.11 ± 9.84	**<0.001**	**<0.001**	**<0.001**
WHR	0.91 ± 0.07	0.93 ± 0.07	0.94 ± 0.08	**<0.001**	**<0.001**	**<0.001**
FPG (mmol/L)	5.17 ± 0.40	5.64 ± 0.57	7.36 ± 2.42	**<0.001**	**<0.001**	**<0.001**
2hPG (mmol/L)	6.01 ± 0.98	8.73 ± 1.15	12.61 ± 4.34	**<0.001**	**<0.001**	**<0.001**
HbA1c (%)	5.64 ± 0.38	5.91 ± 0.45	7.00 ± 1.48	**<0.001**	**<0.001**	**<0.001**
TG (mmol/L)	1.09 (0.82-1.54)	1.32 (0.96-1.85)	1.36 (0.98-2.00)	**<0.001**	**<0.001**	**<0.001**
TC (mmol/L)	4.69 ± 0.96	4.80 ± 1.00	4.76 ± 1.07	**<0.001**	**<0.001**	**0.032**
LDL-C (mmol/L)	2.69 ± 0.75	2.74 ± 0.76	2.72 ± 0.82	0.082	**—**	**—**
HDL-C (mmol/L)	1.34 ± 0.33	1.29 ± 0.34	1.24 ± 0.32	**<0.001**	**<0.001**	**<0.001**
FINS (*μ*U/mL)	5.50 (4.10-7.50)	6.40 (4.60-8.70)	6.90 (4.70-10.10)	**<0.001**	**<0.001**	**<0.001**
HOMA-IR	1.26 (0.92-1.75)	1.60 (1.12-2.22)	2.15 (1.39-3.35)	**<0.001**	**<0.001**	**<0.001**
HOMA-*β*	57.02 (49.41-92.06)	51.26 (43.63-86.06)	43.52 (27.02-69.69)	**<0.001**	**<0.001**	**<0.001**
SBP (mmHg)	126.75 ± 21.51	133.19 ± 21.44	133.44 ± 20.55	**<0.001**	**<0.001**	**<0.001**
DBP (mmHg)	76.09 ± 13.31	77.82 ± 12.92	76.87 ± 12.44	**<0.001**	**<0.001**	0.080
Educational level						
Low (%)	3216 (73.1%)	1328 (71.2%)	1291 (66.8%)	—	—	—
Medium (%)	960 (21.8%)	389 (20.9%)	439 (22.7%)	—	—	—
High (%)	223 (5.1%)	147 (7.9%)	203 (10.5%)	—	—	—
Current drinker (%)	1101 (25.0%)	487 (26.1%)	469 (24.3%)	0.411	—	—
Physical activity (%)	636 (14.5%)	315 (16.8%)	388 (20.1%)	**<0.001**	**0.042**	**<0.001**
Family history of DM (%)	477 (10.8%)	236 (12.7%)	352 (18.2%)	**<0.001**	0.114	**<0.001**
Smoking status						
Never-smoker (%)	3443 (78.3%)	1495 (80.2%)	1524 (78.8%)	—	—	—
Exsmoker (%)	124 (2.8%)	74 (4.0%)	94 (4.9%)	—	—	—
Current smoker (%)	832 (18.9%)	295 (15.8%)	315 (16.3%)	—	—	—
Nonfatal stroke (%)	65 (1.5%)	41 (2.2%)	82 (4.2%)	**<0.001**	0.129	**<0.001**

Data are means ± SD, *n* (%), and median (25th and 75th percentiles). The *P* values were corrected with the Bonferroni correction by multiple comparison between each status of glucose metabolism and were statistically significant at *P* < 0.05 (two-sided). The *P* values considered statistically significant were shown in bold.

**Table 2:** Association of smoking status with T2DM (*N* = 6332).

	Model 1		Model 2		Model 3	
	OR	*P*	OR	*P*	OR	*P*
Smoking status						
Never-smoker	1		1		1	
Exsmoker	**1.71 (1.30-2.25)**	**<0.001**	0.80 (0.71-1.33)	0.848	0.97 (0.70-1.33)	0.847
Current smoker	**0.86 (0.74-0.99)**	**0.032**	**0.79 (0.65-0.97)**	**0.021**	**0.80 (0.66-0.98)**	**0.028**
Daily consumption^∗^						
Never-smoker	1		1		1	
1-19 cigarettes/day	0.92 (0.76-1.12)	0.419	**0.77 (0.60-0.98)**	**0.033**	**0.77 (0.60-0.98)**	**0.033**
≥20 cigarettes/day	**0.80 (0.66-0.97)**	**0.021**	0.80 (0.63-1.01)	0.059	0.79 (0.62-1.01)	0.057
Smoking duration^∗^						
Never-smoker	1		1		1	
<20 years	0.87 (0.70-1.09)	0.222	**0.74 (0.57-0.96)**	**0.024**	**0.64 (0.47-0.87)**	**0.005**
≥20 years	1.01 (0.87-1.18)	0.870	0.87 (0.71-1.06)	0.170	0.84 (0.68-1.04)	0.106
Pack-year^∗^						
Never-smoker	1		1		1	
1-19 pack-years	**0.81 (0.66-0.98)**	**0.031**	**0.75 (0.59-0.95)**	**0.018**	**0.74 (0.59-0.95)**	**0.015**
≥20 pack-years	0.91 (0.75-1.10)	0.307	0.82 (0.65-1.04)	0.103	0.82 (0.65-1.05)	0.112

Model 1: no adjusted variables. Model 2: adjusted for age, BMI, gender, educational level, physical activity, alcohol consumption, and family history of diabetes. Model 3: adjusted for Model 2 plus SBP, DBP, TG, TC, HDL-C, and LDL-C. The sample size was 6332 in the analysis of the subgroup of smoking status. ^∗^Exsmokers were not included in the analysis of the subgroup of daily consumption, smoking duration, and pack-year (*n* = 6144).

**Table 3:** Association between cigarette smoking and T2DM in men stratified by obesity (*N* = 1926).

Subgroup	Model 1		Model 2		Model 3	
	OR	*P*	OR	*P*	OR	*P*
BMI < 25 kg/m^2^						
Never-smoker	1		1		1	
1-19 pack-years	**0.39 (0.28-0.54)**	**<0.001**	**0.46 (0.32-0.66)**	**<0.001**	**0.44 (0.30-0.63)**	**<0.001**
≥20 pack-years	**0.42 (0.31-0.59)**	**<0.001**	**0.47 (0.33-0.67)**	**<0.001**	**0.45 (0.32-0.64)**	**<0.001**
BMI ≥ 25 kg/m^2^						
Never-smoker	1		1		1	
1-19 pack-years	**0.65 (0.45-0.92)**	**0.016**	0.88 (0.60-1.29)	0.524	0.87 (0.59-1.27)	0.467
≥20 pack-years	0.74 (0.54-1.03)	0.072	1.03 (0.72-1.48)	0.856	1.01 (0.71-1.44)	0.959
WC < 90 cm						
Never-smoker	1		1		1	
1-19 pack-years	**0.40 (0.28-0.56)**	**<0.001**	**0.50 (0.34-0.73)**	**<0.001**	**0.47 (0.32-0.69)**	**<0.001**
≥20 pack-years	**0.56 (0.40-0.78)**	**0.001**	**0.69 (0.48-0.99)**	**0.047**	**0.63 (0.44-0.91)**	**0.013**
WC ≥ 90 cm						
Never-smoker	1		1		1	
1-19 pack-years	**0.60 (0.44-0.84)**	**0.003**	0.80 (0.56-1.14)	0.223	0.81 (0.57-1.16)	0.257
≥20 pack-years	**0.56 (0.41-0.76)**	**<0.001**	0.73 (0.52-1.02)	0.065	0.72 (0.51-1.01)	0.060
WHR < 1.0						
Never-smoker	1		1		1	
1-19 pack-years	**0.47 (0.36-0.60)**	**<0.001**	**0.61 (0.46-0.81)**	**0.001**	**0.59 (0.45-0.78)**	**<0.001**
≥20 pack-years	**0.58 (0.46-0.74)**	**<0.001**	**0.75 (0.58-0.98)**	**0.033**	**0.71 (0.54-0.92)**	**0.010**
WHR ≥ 1.0						
Never-smoker	1		1		1	
1-19 pack-years	0.72 (0.38-1.37)	0.312	0.85 (0.43-1.69)	0.639	0.79 (0.37-1.68)	0.534
≥20 pack-years	**0.37 (0.18-0.76)**	**0.007**	0.47 (0.22-1.03)	0.059	0.45 (0.20-1.01)	0.052

Model 1: no adjusted variables. Model 2: adjusted for age, BMI, gender, educational level, physical activity, alcohol consumption, and family history of diabetes. Model 3: adjusted for Model 2 plus SBP, DBP, TG, TC, HDL-C, and LDL-C.

**Table 4:** Interaction between diabetes and smoking in relation to stroke (*N* = 7904).

Smoking	T2DM	Model 1	*P*	Model 2	*P*	Model 3	*P*
No	No	1		1		1	
No	Yes	1.81 (1.28-2.55)	0.001	1.91 (1.35-2.70)	<0.001	1.88 (1.33-2.66)	<0.001
Yes	No	1.90 (1.06-3.43)	0.032	1.87 (1.02-3.45)	0.042	1.85 (1.01-3.41)	0.047
Yes	Yes	2.50 (1.20-5.21)	0.014	2.67 (1.27-5.65)	0.010	2.64 (1.25-5.57)	0.011

Model 1: no adjusted variables. Model 2: adjusted for age, BMI, gender, educational level, physical activity, and alcohol consumption. Model 3: adjusted for Model 2 plus SBP, DBP, TG, TC, HDL-C, and LDL-C.

## Data Availability

The data are held in a secure, confidential database, which can only be assessed by members of the REACTION group. The REACTION has a website (http://www.rjh.com.cn/pages/neifenmike/REACTION/index.shtml).

## Supplementary Materials

Supplementary Materials Supplementary Table 1: characteristics of the population according to the status of glucose metabolism (*N* = 8196). Supplementary Table 2: association of smoking status with prediabetes (*N* = 6263). Supplementary Table 3: association between cigarette smoking and prediabetes in men stratified by obesity (*N* = 1846).
