# Supplementary material for: Cigarette Smoking Is Negatively Associated with the Prevalence of Type 2 Diabetes in Middle-Aged Men with Normal Weight but Positively Associated with Stroke in Men
Source: J Diabetes Res. 2019 Sep 11;2019:1853018. doi: 10.1155/2019/1853018 (PMC6755302; doi:10.1155/2019/1853018)
Supplement: Supplementary Materials — Supplementary Table 1: characteristics of the population according to the status of glucose metabolism (N = 8196). Supplementary Table 2: association of smoking status with prediabetes (N = 6263). Supplementary Table 3: association between cigarette smoking and prediabetes in men stratified by obesity (N = 1846). [file 1853018.f1.pdf]

Supplementary Table 1 Characteristics of the population according to the status of glucose metabolism (N=8196)

Variables	Men			Women (D) n=5422	*P*			
	All (A) n=2774	BMI<25kg/m^2 (B) n=1433	BMI≥25kg/m^2 (C) n=1341		B vs. C	B vs. D	C vs. D	A vs. D
Age (years)	59.82±10.69	60.53±10.75	59.04±10.58	58.25±10.25	**<0.001**	**<0.001**	**0.013**	**<0.001**
BMI (kg/m^2)	24.99±3.31	22.53±1.80	27.63±2.41	24.74±3.51	**<0.001**	**<0.001**	**<0.001**	**0.001**
WC (cm)	90.31±8.50	85.74±7.07	95.17±7.02	88.23±9.69	**<0.001**	**<0.001**	**<0.001**	**<0.001**
WHR	0.93±0.06	0.91±0.06	0.95±0.06	0.92±0.08	**<0.001**	**<0.001**	**<0.001**	**<0.001**
FPG (mmol/L)	5.91±1.64	5.85±1.66	5.98±1.60	5.74±1.47	**0.028**	**0.017**	**<0.001**	**<0.001**
2hPG (mmol/L)	8.33±3.68	8.26±3.81	8.40±3.53	8.11±3.45	0.270	0.164	**0.006**	**0.010**
HbA1c (%)	6.05±1.01	5.99±1.00	6.13±1.01	6.00±0.95	**<0.001**	0.468	**<0.001**	**0.040**
TG (mmol/L)	1.19(0.87-1.69)	1.05(0.81-1.51)	1.41(1.02-2.06)	1.22(0.88-1.81)	**<0.001**	**<0.001**	**<0.001**	**0.011**
TC (mmol/L)	4.61±0.96	4.58±0.96	4.62±0.97	4.81±1.01	0.332	**<0.001**	**<0.001**	**<0.001**
LDL-C (mmol/L)	2.66±0.76	2.61±0.75	2.70±0.77	2.74±0.78	**0.003**	**<0.001**	0.091	**<0.001**
HDL-C (mmol/L)	1.24±0.33	1.32±0.36	1.16±0.28	1.34±0.33	**<0.001**	**0.038**	**<0.001**	**<0.001**
FINS(μU/mL)	5.50(3.90-7.90)	4.50(3.30-6.20)	6.90(5.10-9.60)	6.20(4.50-8.60)	**<0.001**	**<0.001**	**<0.001**	**<0.001**
HOMA-IR	1.41(0.95-2.09)	1.11(0.78-1.62)	1.79(1.24-2.52)	1.52(1.06-2.21)	**<0.001**	**<0.001**	0.970	<0.001
HOMA-β SBP	54.38(36.97-80.29)	46.41(31.54-66.15)	65.76(44.37-94.79)	64.20(45.40-89.95)	**<0.001**	**<0.001**	**<0.001**	**<0.001**
(mmHg) DBP	131.60±21.35	129.19±21.40	134.17±21.03	128.87±21.55	**<0.001**	0.622	**<0.001**	**<0.001**
(mmHg)	79.05±13.59	77.10±13.58	81.13±13.29	75.44±12.58	**<0.001**	**<0.001**	**<0.001**	**<0.001**
Smoking status								
never-smoker (%)	1139(41.1%)	568(39.6%)	571(42.6%)	5323(98.2%)	-	-	-	
ex-smoker (%)	280(10.1%)	134(9.4%)	146(10.9%)	12(0.2%)	-	-		
current smoker (%)	1355(48.8%)	731(51.0%)	624(46.5%)	87(1.6%)	-	-	-	
T2DM(%)	741(26.7%)	388(27.1%)	353(26.3%)	1192(22.0%)	0.654	**<0.001**	**0.001**	**<0.001**
nonfatal stroke (%)	67(2.4%)	33(2.3%)	34(2.5%)	121(2.2%)	0.690	0.871	0.506	0.599

Supplementary Table 2 Association of smoking status with pre-diabetes(N=6263)

	Model 1		Model 2		Model 3	
	OR	*P*	*OR*	*P*	*OR*	*P*
Smoking status						
Never-smoker	1		1		1	
Ex-smoker	**1.37(1.02-1.85)**	**0.034**	0.90(0.64-1.24)	0.503	0.91(0.65-1.26)	0.553
Current smoker	**0.82(0.71-0.95)**	**0.007**	**0.75(0.62-0.92)**	**0.005**	**0.77(0.63-0.93)**	**0.008**
Daily consumption						
Never-smoker	1		1		1	
1-19 cigarettes/day	**0.80(0.65-0.98)**	**0.034**	**0.69(0.54-0.88)**	**0.003**	**0.68(0.53-0.88)**	**0.003**
≥20 cigarettes/day	0.83(0.69-1.00)	0.054	**0.78(0.61-0.98)**	**0.038**	**0.78(0.61-0.99)**	**0.040**
Smoking Duration						
Never-smoker	1		1		1	
<20 years	0.84(0.67-1.05)	0.122	**0.73(0.57-0.95)**	**0.018**	**0.69(0.52-0.93)**	**0.016**
≥20 years	0.91(0.78-1.07)	0.260	**0.80(0.65-0.98)**	**0.034**	**0.75(0.60-0.93)**	**0.010**
Pack-year						
Never-smoker	1		1		1	
1-19 pack-years	**0.78(0.64-0.95)**	**0.013**	**0.71(0.56-0.90)**	**0.005**	**0.71(0.54-0.90)**	**0.005**
≥20 pack-years	0.86(0.71-1.04)	0.119	**0.76(0.60-0.97)**	**0.025**	**0.76(0.60-0.98)**	**0.029**

Model 1: No adjusted variables

Model 2: Adjusted for age, BMI, gender, educational level, physical activity, alcohol consumption and family history of diabetes

Model 3: Adjusted for Model 2 plus SBP, DBP, TG, TC, HDL-C and LDL-C

The sample size was 6263 in the analysis of the subgroup of smoking status. * Ex-smokers were not included in the analysis of the subgroup of daily consumption, smoking duration and pack-year (n=6065).

Supplementary Table 3 Association between cigarette smoking and pre-diabetes in men stratified by obesity(N=1846)

Subgroup	Model 1		Model 2		Model 3	
	OR	*P*	*OR*	*P*	*OR*	*P*
BMI<25kg/m^2						
Never-smoker	1		1		1	
1-19 pack-years	**0.52(0.36-0.74)**	**<0.001**	**0.54(0.37-0.80)**	**0.002**	**0.52(0.35-0.77)**	**0.001**
≥20 pack-years	**0.63(0.45-0.88)**	**0.007**	**0.61(0.42-0.89)**	**0.009**	**0.60(0.42-0.87)**	**0.008**
BMI≥25kg/m^2						
Never-smoker	1		1		1	
1-19 pack-years	0.76(0.54-1.08)	0.124	0.94(0.65-1.36)	0.942	0.96(0.66-1.39)	0.824
≥20 pack-years	**0.71(0.51-0.99)**	**0.044**	0.88(0.60-1.29)	0.524	0.89(0.62-1.28)	0.525
WC<90cm						
Never-smoker	1		1		1	
1-19 pack-years	**0.38(0.25-0.58)**	**<0.001**	**0.44(0.28-0.69)**	**<0.001**	**0.42(0.27-0.66)**	**<0.001**
≥20 pack-years	0.70(0.49-1.01)	0.056	0.77(0.52-1.14)	0.196	0.73(0.49-1.08)	0.116
WC≥90cm						
Never-smoker	1		1		1	
1-19 pack-years	0.85(0.62-1.16)	0.310	0.97(0.69-1.36)	0.858	0.99(0.71-1.39)	0.958
≥20 pack-years	**0.64(0.47-0.88)**	**0.006**	0.74(0.53-1.03)	0.076	0.76(0.54-1.07)	0.112
WHR<1.0						
Never-smoker	1		1		1	
1-19 pack-years	**0.54(0.41-0.72)**	**0.001**	**0.63(0.47-0.84)**	**0.002**	**0.62(0.46-0.83)**	**0.001**
≥20 pack-years	**0.66(0.51-0.85)**	**0.001**	**0.73(0.55-0.96)**	**0.024**	**0.72(0.55-0.95)**	**0.020**
WHR≥1.0						
Never-smoker	1		1		1	
1-19 pack-years	1.15(0.63-2.10)	0.656	1.27(0.67-2.40)	0.452	1.30(0.65-2.61)	0.460
≥20 pack-years	0.72(0.39-1.35)	0.311	0.88(0.46-1.71)	0.709	0.84(0.42-1.69)	0.626

Model 1: No adjusted variables

Model 2: Adjusted for age, BMI, gender, educational level, physical activity, alcohol consumption and family history of diabetes

Model 3: Adjusted for Model 2 plus SBP, DBP, TG, TC, HDL-C and LDL-C
